# Outcomes of 72-Hour Hospital Treatment Responsibility Post Discharge to Municipal Nursing Care

**DOI:** 10.5334/ijic.10188

**Published:** 2026-09-07

**Authors:** Elise Harder, Ann-Sofie Kofoed Berthelsen, Julie Christina Grew, Sidsel Maria Jørgensen, Fiona Haustedt Mossman, Michaela Louise Schiøtz

**Affiliations:** 1 Center for Clinical Research and Prevention, Copenhagen University Hospital, Frederiksberg, Denmark; 2 Department of Public Health, University of Copenhagen, Copenhagen, Denmark; 3 Research Unit of General Practice, Department of Public Health, University of Southern Denmark, Odense, Denmark

**Keywords:** cross-sectoral collaboration, treatment quality, cross-sector transition, patient pathways, integrated care

## Abstract

**Background:**

The increasing prevalence of individuals living with chronic conditions and multi-morbidity presents challenges to healthcare systems and coordination of coherent patient pathways. In Denmark, transitions between healthcare sectors are susceptible to disruption due to inadequate cross-sectoral collaboration. To improve continuity of care and cross-sectoral collaboration, an agreement of 72-hour hospital treatment responsibility post-discharge was implemented.

**Methods:**

We used data from a questionnaire survey for healthcare professionals, data from electronic patient records, and data from cross-sectoral medical record audits to evaluate outcomes of the implementation of the agreement.

**Results:**

Survey data suggested that the agreement may enhance cross-sectoral collaboration by improving communication and understanding of other sectors, with municipal healthcare professionals perceiving these benefits more strongly. Data from electronic patient records indicated increased attention to discharge documentation. Medical record audits suggest that the agreement may be supportive where medication-related uncertainties necessitated clarification.

**Conclusion:**

The evaluation shows that the agreement can facilitate cross-sectoral communication, in some cases may improve treatment quality and safety and may support safer transitions from hospital to municipal care. The agreement does not reduce readmissions among the target group in general; however, it might facilitate avoidance of unnecessary readmissions in cases where cross-sectoral dialogue is sufficient.

## Introduction

### Background

The world’s demographic composition is undergoing significant transformations, resulting in a growing proportion of elderly and an associated rise in chronic diseases [1]. Altogether, this causes considerable pressure on healthcare systems’ resources and capacity [2]. This demographic shift presents a challenge in organising coherent patient pathways, as the complexity of treatment and care requirements intensifies [3].

In the context of the Danish healthcare system, characterized by a division of responsibilities between hospitals and municipal care providers, these challenges are particularly pronounced [4]. The transition from hospital to municipal care represents a vulnerable point in the healthcare system, and continuity of patient care is often challenged [5,6,7], potentially resulting in diminished quality of treatment and hospital readmissions [7,8]. Several factors contributing to this critical transition phase have been identified, including insufficient communication and information-sharing [4,9,10,11,12,13], and lack of collaboration between sectors [10,13,14,15,16].

Transitional care has been defined as a set of actions designed to ensure coordination and continuity of healthcare as patients transfer between different locations or levels of care [17]. Several transitional care models have been proposed, highlighting the importance of cross-sectoral communication, involvement of patients, and coordinated follow-up to secure safe transition [18,19]. In 2022, an agreement introducing a 72-hour treatment responsibility for the somatic hospitals in the Capital Region of Denmark was established, reflecting principles found in existing transitional care models. Some European countries have introduced alternative ways of organizing access to specialist care, for example through patient-initiated follow-up [20] and self-referral [21]. In Italy, Territorial Operative Centers (COTs) are designed to coordinate patient care and ensure linkage between services and professionals across different care settings. It supports transitions from hospital to community-based care and aims to reduce avoidable readmissions as well as inappropriate use of emergency department services [22]. The 72-hour treatment responsibility agreement distinguishes itself from these initiatives by establishing a clear communication pathway directly between the hospitals and municipalities. It mandates that hospitals provide a direct telephone number, thereby enhancing the ability of municipalities to receive timely and qualified support when necessary. Due to limited knowledge and no evidence regarding the effects of implementing this specific agreement implemented in a Danish context, we aimed to evaluate outcomes of the implementation of a 72-hour treatment responsibility agreement for hospitals with a specific focus on cross-sectoral collaboration, treatment quality and safety in the transition between hospital and municipality, and hospital readmissions.

## Methods

### Setting

The implementation was conducted in the Capital Region of Denmark, including six somatic hospitals, 29 municipalities and all general practices within the region. Data collection took place from February 2022 throughout December 2023. To support the implementation of the agreement, a steering group consisting of patient representatives, representatives from hospitals, municipalities, and general practitioners (GPs) was established. The steering group was responsible for the implementation, monitoring, and evaluation of the agreement.

### Intervention

**Figure 1:**
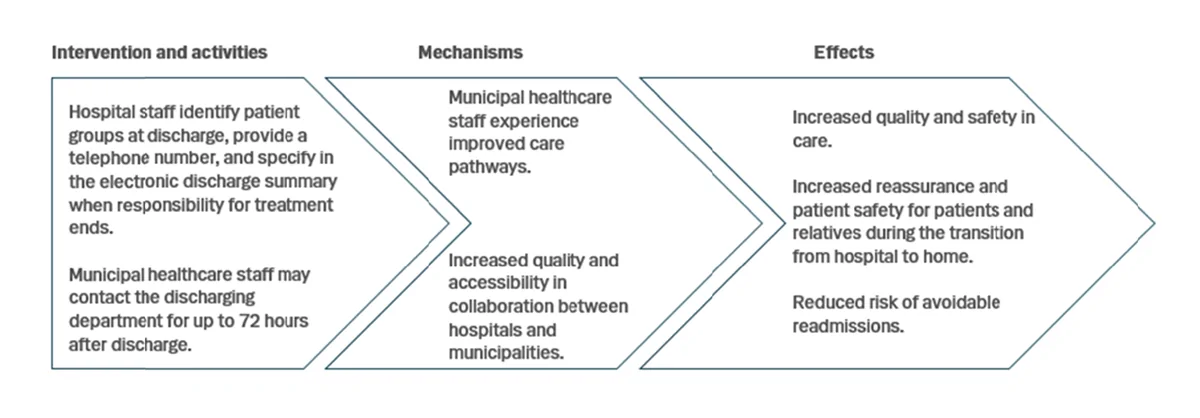
Logic model of the 72-hour treatment responsibility for hospitals.

The 72-hour treatment responsibility agreement aims to improve the transition from hospital to municipality for the target group consisting of patients hospitalized for at least 24 hours and discharged to municipal nursing care, i.e. temporary care facilities, nursing homes or nursing care provided in citizens’ own homes. Specifically, the 72-hour treatment responsibility agreement gives municipal healthcare professionals the opportunity to contact the hospital for assistance in situations where uncertainty arises regarding the patient’s care plan or health status. The intervention can be described through a logic model as illustrated in Figure 1. Figure 1 shows how the intervention is expected to improve the transition from hospital to care in the municipalities. Healthcare professionals at the hospitals identify relevant patient groups at discharge, provide a phone number for the discharging department, and indicate in the electronic discharge papers when clinical responsibility ends. This allows municipal healthcare professionals to contact the discharging department within 72 hours after discharge in case of any uncertainties related to the patient’s health condition. The mechanism in this intervention is expected to enhance coordination and accessibility in the hospital–municipality collaboration as well as to give municipal healthcare professionals an experience of a more coherent patient pathway. In the long term, this is expected to contribute to improved treatment quality and safety, higher levels of perceived safety among patients and relatives during the transition from hospital to home, and a reduced risk of unnecessary readmissions.

While this paper focuses on the outcomes of this agreement, elements of importance for the implementation of the agreement has been described elsewhere (Berthelsen et al., unpublished results).

### Study Design

To evaluate the implementation of a 72-hour treatment responsibility a combination of quantitative and qualitative data was used. A questionnaire survey was conducted among healthcare professionals from both hospitals, municipalities, and general practice to gain knowledge about the perception and use of the agreement among those working with the agreement in daily clinical practice. To examine challenges in the patient pathway in relation to the use of the 72-hour treatment responsibility agreement, cross-sectoral medical record audits of selected patient cases covered by the 72-hour treatment responsibility were conducted. Finally, quantitative data from electronic patient records concerning all patients covered by the agreement was extracted to investigate overall characteristics of the patients, communication activity between hospitals and municipalities, and readmissions among the target group. Data from electronic patient records was derived from the Health Platform (in Danish: Sundhedsplatformen), which is an electronic health record system used in Eastern Denmark. The system supports management of patient data, clinical records, medication, and care processes in Eastern Denmark. This approach facilitated a nuanced analysis of the research topics and allowed the alignment of quantitative findings with subjective perspectives. The reporting of the study was inspired by the Consensus-Based Checklist for Reporting of Survey Studies (CROSS) [23] due to the inclusion of survey data.

### Study populations, data collection methods, and data analysis

#### Survey

The study population for the survey was hospital and municipal healthcare professionals, and GPs experienced in working with the agreement in their everyday clinical practice. We aimed to invite 200 healthcare professionals from each of the three healthcare sectors. In total, 194 healthcare professionals from five hospitals, 223 healthcare professionals from eight municipalities, and 200 general practices were invited.

The survey was developed in collaboration with a working group consisting of representatives from hospitals, municipalities, and general practice who volunteered to help qualifying the questions. This process resulted in two surveys: one for hospitals and municipalities, and a shorter version for GPs. The GPs received a shorter version as the working group assessed that GPs were not involved in the daily workflow of the agreement to the same extent as hospital and municipal healthcare professionals.

Themes in the questionnaires included implementation and operation of the agreement, perceived meaningfulness of the agreement, use of the agreement, cross-sectoral collaboration, and impact on patient pathways. In the present study, only questions concerning perceived meaningfulness, cross-sectoral collaboration, and impact on patient pathways are included. Table 1 provides a detailed overview of data collected with each data collection method and a description of the data.

**Table 1:** Overview of data collection methods and a brief description of data.


DATA SOURCE	DESCRIPTION

Survey	Survey for municipalities and hospitals: 18 questions Survey for GPs: five questions Themes included: - Implementation and operation of the agreement - Perceived meaningfulness of the agreement - Use of the agreement - Cross-sector collaboration - Impact on patient pathways Both questionnaires were distributed by email. Municipalities and hospitals received a reminder after one week with no answer. To remind GPs, a description of the survey was published on a professional website for GPs.

Cross-sectoral medical record audits	Audit panels had the possibility to look up necessary information on a case in local electronic systems. Focus at the meetings was on application of the agreement, cross-sectoral collaboration and communication, and prevention of readmissions.

Electronic patient records	

Number of discharge reports sent	Upon discharge to municipal nursing care, hospitals must send discharge reports to the municipality. These reports include essential information on the patient’s care plan. The agreement enabled indicating in the reports if the patient was discharged with a 72-hour treatment responsibility. Fluctuations in the number of discharge reports issued could suggest a pronounced focus on discharge documentation with the agreement. We compared the number of discharge reports with the number of discharge reports in the target group in 2023.

Number of correspondence messages sent and received	Correspondence messages are a secure way of electronic communication within the healthcare sector. The agreement stipulates that telephone communication between the municipality and the hospital must be supplemented by written correspondence from the hospital to the municipality. Fluctuation in corresponding messages sent and received was seen as an indication of the cross-sectoral communication about patients.

Number of updated shared medication records (Fælles Medicinkort) at discharge	The shared medication record is a national IT solution in the Danish healthcare system, designed to provide an overview of each citizen’s current medication. Updated shared medication record at discharge is one aspect of a secure transition between sectors. We investigated the percentage of patients discharged with 72-hour treatment responsibility having updated shared medication records. This number was compared with the corresponding number among all individuals discharged.

Number of readmissions within 30 days after discharge among citizens covered by the agreement	We investigated the development in readmissions in the target group to get insights in the agreement’s potential to prevent readmissions.Readmissions were characterized as a hospital stay lasting 12 hours or more within 30 days following the initial admission, accompanied by a 72-hour treatment responsibility.Admissions related to cancer or cancer-associated conditions; hospice stays; or readmissions resulting from accidents, violence, or suicide attempts were excluded.


Table describes the three data collection methods used for the study together with a brief description of the collected data. GPs: General practitioners.

Healthcare professionals from hospitals were recruited through a group of regional representatives who had followed the implementation of the agreement. Healthcare professionals from municipalities were recruited via contact to the municipalities, and GPs were recruited through a random selection of licence numbers. The survey was conducted over a two-month period from the beginning of November 2023 to the end of December 2023.

Based on responses from the questionnaires, descriptive statistical analysis of overall response rates and response rates on closed-ended questions were conducted. The questionnaires contained five response categories, i.e. ‘strongly agree’, ‘agree’, ‘not relevant’, ‘disagree’ and ‘strongly disagree’. In the analyses, the response categories ‘strongly agree’ and ‘agree’ were combined and the response categories ‘disagree’ and ‘strongly disagree’ were combined. The analyses were conducted with R software, version 4.3.0 (R Core Team 2023). Using inspiration from a thematic analysis approach [24], text from open-ended questions were extracted and analysed. Themes often occurring in the data were identified and used to nuance quantitative findings.

#### Cross-sectoral medical record audits

Altogether, 35 patient cases from 24-hour temporary care facilities, nursing homes, and nursing care in citizens’ own homes were included in cross-sectoral medical record audits. For a case to be relevant for auditing, there should have been at least one phone call between hospital and municipality within 72 hours post discharge. Audits were conducted by audit panels consisting of two healthcare professionals from hospitals and two to three healthcare professionals from municipalities.

Before each audit meeting, the members of the audit panel had prepared the patient cases to be discussed using local IT documentation systems. During the audit meetings, the panel went through a structured form with questions related to the use of the agreement. At the meetings, the audit panel filled in one final structured form for each patient case. The audit meetings were audio-recorded and transcribed.

Data from the medical record audits were analysed using counting of relevant answers. Moreover, short patient case descriptions were written for each patient case providing information on characteristics of the cases that underwent auditing.

#### Electronic patient records

Data from electronic patient records on all individuals discharged with coverage by 72-hours treatment responsibility were extracted from September 2022, where the entire target group was enrolled in the intervention, until ultimo December 2023.

To assess activities such as digital cross-sectoral communication during the 72-hour treatment responsibility period and immediately thereafter as well as potential readmissions, indicators for these measures were extracted from electronic patient records. Descriptive statistical analyses were conducted on extracted data.

### Involvement of people with lived experience

Representatives with lived experience were involved throughout the development and implementation of the 72-hour agreement as members of the steering group. In the steering group they contributed with their perspectives to discussions. We did not include people with lived experience in the preparation of this paper.

### Ethical considerations

The study complies with the requirements of the European Union General Data Protection Regulation. According to Danish legislation, this study did not require approval from a research ethics committee, as it consists of questionnaires and interviews without biological sampling. Moreover, quality improvement projects and quality control do not require approval from a research ethics committee. Instead, the project is reported in the regional electronic research registration system (J. nr. P - 2022–24). Reporting research projects in this system ensures compliance with GDPR requirements, and that appropriate approvals and data protection are in place. All healthcare professionals participating in the survey and all patients whose case underwent audit received thorough information about the project and provided written informed consent to participate.

## Results

### Characteristics of study populations

#### Survey

The study population for the survey was healthcare professionals across hospitals, municipalities, and general practice. Table 2 presents response rates distributed between sectors. Close to one fourth of the invited GPs responded to the questionnaire. Among municipal healthcare professionals and hospital healthcare professionals, one third and half of invitees responded, respectively. Tables showing primary questions in the questionnaires and responses to the survey is provided in Supplementary 1.

**Table 2:** Response rates to the survey distributed on sectors.


CHARACTERISTICS	n (%)

General practitioners	48 (24)

Municipal healthcare professionals	73 (33)

Hospital healthcare professionals	95 (49)


Table shows number and percentage of invited healthcare professionals in each sector responding to the survey distributed on General practitioners, municipal healthcare professionals and hospital healthcare professionals. n: number of responders.

#### Cross-sectoral medical record audit

The patient cases undergoing cross-sectoral medical record audit were very similar across 24-hour temporary care facilities, nursing homes and municipal nursing care in citizens’ homes in relation to age span and main reasons for hospitalization, especially including chronic diseases such as COPD, cancer, and heart disease; lung infections; and general frailty (Table 3). For an overview of the questions used for guidance at the audit meetings, please see Supplementary 2.

**Table 3:** Characteristics of the study population for cross-sectoral medical record audits.


CHARACTERISTICS	24-HOUR TEMPORARY CARE FACILITIES	NURSING HOMES	OWN HOMES

Number of cases	20	7	8

Gender			

Women	7	5	3

Age span (years)	60–95	68–93	59–88

Main reasons for hospital admission	Fall Fracture COPD Lung infection Confusion Heart disease Cancer Covid-19 General frailty	Fall Fracture COPD Lung infection Confusion Depression Anxiety General frailty	Fall Heart disease Diabetes type 2 Diarrhoea Kidney problems Back pain General frailty


Table shows the characteristics of the study population for cross-sectoral medical record audits conducted on patient cases from 24-hour temporary care facilities, nursing homes, and cases where municipal nursing care is provided in the citizens’ own homes. COPD: Chronic Obstructive Pulmonary Disease.

#### Electronic patient records

The number of citizens enrolled under the agreement stabilized on approximately 2,000 per month from September 2022 where the agreement was fully implemented to December 2023. One citizen can have more than one admission and therefore possibly enrolled under the agreement multiple times. Available characteristics of all citizens covered by the agreement showed an overrepresentation of individuals aged 70–89 years and a slight predominance of women (55%). Supplementary 3 contains data showing progression in relevant indicators derived from the Health Platform.

### Cross-sector collaboration

Overall, survey data indicate that healthcare professionals across the three sectors generally perceive the hospitals’ 72-hour post-discharge treatment responsibility for patients in the target group as meaningful. Among GPs, municipal healthcare professionals, and hospital healthcare professionals, 96%, 97% and 75%, respectively, confirmed that the hospital’s prolonged treatment responsibility for this patient group is meaningful.

#### Dialogue and communication across sectors

Survey data indicate that healthcare professionals perceive that the agreement has the potential to enhance patient-related dialogue across sectors and to support cross-sectoral communication about discharged patients. The majority (72%) of municipal healthcare professionals stated that the agreement enhances patient-related dialogue, while a little less than half (46%) of hospital healthcare professionals shared this view. One frequently mentioned reason in open-ended questions for why hospital healthcare professionals do not perceive an improvement in communication is that they find several of the phone calls received during the 72 hours to be irrelevant to them.

Survey data also suggest that healthcare professionals perceive that the agreement has led to changes in cross-sector communication, particularly among municipal healthcare professionals. A substantial proportion (65%) of municipal healthcare professionals reported that they have modified their cross-sectoral communication practices, whereas this was only the case for 26% of hospital healthcare professionals. Among GPs, 43% reported changes in their cross-sectoral communication practices. Furthermore, half of the municipal healthcare professionals agreed that the agreement increases frequency of cross-sectoral communication, while one quarter of hospital healthcare professionals agreed on this.

Healthcare professionals from hospital and municipalities were asked if the agreement had facilitated a better understanding of the working conditions of colleagues in other sectors. In total, 39% of municipal healthcare professionals and 26% of hospital healthcare professionals agreed on this.

#### Electronic communication and information sharing

Data extracted from electronic patient records show that a discharge report was provided for 82% of patients who were discharged and covered by the 72-hour treatment responsibility agreement. Regarding the use of digital correspondence messages, a secure way of electronic communication within the healthcare sector, messages were sent from hospitals to municipalities or GPs for 16-17% of patients who were discharged and covered by the agreement. Moreover, data from medical record audits revealed cases where relevant correspondence messages were not sent between sectors, indicating a potential deficiency in the communication.

### Quality of care and safety

Findings from cross-sectoral medical record audits indicate that the agreement supports quality of care in some cases by facilitating identification of errors and misunderstandings. Of the 35 patient cases examined, the audit panels found that treatment quality was improved due to the agreement in 15 cases. The audit panels noted that the quality improvement was especially related to cases where clarification of uncertainties regarding medication was necessary. Moreover, data from electronic patient records show that the shared medication records, a national, digital overview of a citizen’s current and previously prescribed medications, were updated upon discharge in almost 100% of the cases.

### Coherent patient pathways

Survey data show that 56% of healthcare professionals in municipalities and 42% of healthcare professionals in hospitals perceived the 72-hour treatment responsibility agreement to improve the transfer of patients from hospital to municipal care. Additionally, 74% of municipal healthcare professionals agreed that the agreement promotes coherent patient pathways, while 46% of hospital healthcare professionals shared this view.

### Hospital readmissions

Data from electronic patient records show that the readmission rate in the target population has remained consistent around 24% since the entire target group was enrolled in the intervention in 2022. Readmissions were defined as a hospital stay lasting 12 hours or more within 30 days following the initial admission. Among the 35 audited patient cases, we had information on 16 readmissions, of which 12 readmissions happened within 72 hours post discharge. In all 16 cases of readmission, the audit panels assessed the readmissions as not preventable, emphasizing the target group’s vulnerability and need for frequent hospital admission. Among the 35 audit cases, the audit panels found that the agreement may have prevented unnecessary readmissions in five cases, for example due to crucial dialogue across sectors.

## Discussion

This study evaluated outcomes of a 72-hour treatment responsibility agreement between somatic hospitals, municipalities, and GPs in the Capital Region of Denmark. Using several data sources, the results indicate that the agreement can facilitate collaboration between healthcare professionals across sectors, promote coherent patient pathways, and may support structures for enhanced treatment quality and safety. The results underline that the target group represents a vulnerable population where hospital readmissions are often necessary.

Healthcare professionals across municipalities, hospitals, and GPs reported changes in communication across sectors after implementation of the agreement, and half of municipal healthcare professionals and one fourth of hospital healthcare professionals reported increased communication with colleagues from other sectors. While modified and increased communication does not inherently lead to better communication, these changes suggest that the agreement supports structures facilitating communication about patients across sectors. Moreover, most municipal healthcare professionals reported that the agreement improves patient-related dialogue, whereas less than half of hospital healthcare professionals shared this view. These differences in perceived improvements in communication across sectors is likely due to the agreement’s structure, which gives municipalities a clearer pathway to engage with hospitals that are obliged to be on disposal with their expertise.

Authors of a previous overview of reviews [11] find that facilitators for providing cross-sector services are clarity of goals and purpose of arrangements. Based on this, to achieve a more equal perception of collaboration beneficials of the agreement, identifying more relevant and clearer overall goals of the agreement for healthcare professionals at the hospitals might be a solution in the future work with implementing the agreement nationally.

Several studies describe the importance of sharing adequate information in the transition phase between primary and secondary healthcare sectors [12,14,25]. Moreover, the literature describes how delays and omissions in communication often occur [9,10,11], which might result in a decreased level of post discharge care [12,26,27]. In this context, the 72-hour agreement complies with existing knowledge about the importance of communication between professionals to provide high-quality transitional care [17] and can potentially support structures for this crucial information exchange between sectors by providing an established communication pathway. This is a vital aspect of transitional care, where effective communication between the parties involved in sending and receiving is essential for successful care transitions [19]. Sharing of knowledge about patients might contribute to a best-case scenario where healthcare providers across sectors complement each other professionally, facilitating a safe transition for the patients [28]. In line with this, studies of models aiming to support coordination of care for frail and chronic patients across sectors, such as the Family or Community Nurses (FCNs) in Italy, can be pivotal with regard to enhancing care in this group [29].

The implementation of the agreement seems to have put a more pronounced focus on digital communication documents, for example discharge reports and shared medication records. In total, 82% of patients discharged covered by the agreement had a discharge report. In comparison, data from a national database containing data on primary and community healthcare show that a little less than 50% of the discharge reports sent by hospitals had a corresponding admission report from the municipalities in 2022. Moreover, close to 100% among patients discharged covered by the agreement had an updated shared medication record. In comparison, the number is 80% among all patients discharged from hospitals.

A Danish study of cross-sectoral challenges in collaboration within mental health service [10] describes how lack of mutual understanding of each other’s work across municipality settings and hospital settings has pivotal consequences for collaboration. In the present study, we found that particularly many of municipal healthcare professionals agreed that the agreement improves understanding of the work of colleagues in other sectors, suggesting that implementing formal agreements of this type might support overcoming such barriers.

There is a notable difference in how municipal and hospital healthcare professionals view the agreement’s impact on coherent patient pathways. The disparity suggests that the agreement may have varying effects in the different healthcare sectors or is at different implementation stages in the two sectors. This calls for more research in long-term outcomes of the implementation of the agreement and for an evaluation of the ongoing, nationwide implementation. Despite this disparity, the implementation strategy, in which the agreement was formalized as a binding task and supported by regional endorsement, appeared to be an appropriate approach for promoting the implementation of initiatives in the healthcare system.

### Strengths and limitations

One of the principal strengths of the study is the integration of multiple qualitative and quantitative data sources. This methodological approach facilitates a more nuanced analysis of the research topics and allows for the alignment of quantitative findings with healthcare professionals’ perspectives. Another significant strength is the employment of questionnaire data. The use of surveys enables the collection of a substantial volume of quantifiable data, which is useful in identifying and analysing trends and patterns in healthcare professionals’ perceptions regarding the agreement. This methodological choice provides a robust foundation for assessing the agreement’s impact from the perspectives of those directly engaged with its implementation. However, the study also has some limitations. Data from electronic patient records may be incomplete, as documentation is sometimes deprioritized in busy clinical settings, potentially affecting the accuracy of selected measures. Additionally, the limited number of cases (n = 35) included in the cross-sectoral medical record audits may constrain the extent to which the findings can be generalized. As a result, the outcomes from this part should be interpreted with caution. Nevertheless, within the multi-method approach, the analysis still offers contextual insights that enhance the other data sources in the research.

Respondents in the questionnaire survey for hospitals and municipalities may not represent the entire population of healthcare professionals due to the recruitment process, wherein email addresses were provided by contact persons. This method may have introduced selection bias. Moreover, the overall response rates were low for both healthcare professionals in municipalities and hospitals and among GPs, potentially resulting in a skewed depiction of the agreement compared with the actual situation. In data available, it was not possible to identify who received municipal nursing care before the agreement was implemented, preventing investigating if the number of readmissions is reduced post implementation of the agreement. Instead, the trend in the number of readmissions in the target group since the implementation of the agreement was examined. The response categories in the questionnaires were limited to indicating whether healthcare professionals agreed or disagreed with a given statement. Consequently, we were unable to ascertain if healthcare professionals held a contrary opinion to the statement. Finally, as the agreement was developed within the specific context of the Danish healthcare system, the generalizability of findings to other countries may be limited.

In this study, we did not incorporate patient perspectives on the agreement. This decision was made because the agreement operates at the organizational level and patients in the target group are not necessarily made aware of its existence.

### Future perspectives

The 72-hour hospital treatment responsibility agreement is currently being implemented in all Danish regions providing the opportunity to evaluate its outcomes on a larger scale in the near future. Moreover, The Capital Region of Denmark has extended the treatment responsibility to 96 hours and expanded the target group to include patients discharged from psychiatric care. It will be relevant to evaluate the potential effects of this extension in order to illuminate both clinical and organizational implications.

## Conclusion

Overall, the evaluation of the implementation of the 72-hour hospital treatment responsibility agreement shows that the agreement can facilitate cross-sectoral communication. Digital communication data suggest an increased attention to discharge documentation due to the agreement. Moreover, the agreement may improve quality of care in cases involving medication-related uncertainty and may supports safer transitions from hospital to municipal care by reducing the risk of errors. Finally, the agreement does not appear to reduce readmissions among the target group in general; however, it might facilitate avoidance of unnecessary readmissions in cases where cross-sectoral dialogue is sufficient.

## Additional Files

The additional files for this article can be found as follows:

10.5334/ijic.10188.s1 Supplementary 1. Distribution of responses to primary questions in the survey.

10.5334/ijic.10188.s2 Supplementary 2. Questions used for guidance at the audit meetings.

10.5334/ijic.10188.s3 Supplementary 3. Progression in indicators from electronic patient records derived from the Health Platform.
