# Supplementary material for: Outcomes of 72-Hour Hospital Treatment Responsibility Post Discharge to Municipal Nursing Care
Source: Int J Integr Care. 2026 Sep 7;26(3):15. doi: 10.5334/ijic.10188 (PMC13564373; doi:10.5334/ijic.10188)
Supplement: Supplementary file 1 [file ijic-26-3-10188-s1.pdf]

Supplementary 1

Distribution of responses to primary questions in the survey

	Agree n (%)			Disagree n (%)		
Question	Hospital	Municipal	GP	Hospital	Municipal	GP
1	71 (75)	71 (97)	46 (96)	24 (25)	2 (2)	1 (2)
2	74 (78)	58 (80)	27 (56)	18 (19)	15 (20)	21 (44)
3	74 (78)	56 (69)	NA	21 (22)	12 (16)	NA
4	81 (85)	65 (89)	32 (67)	12 (12)	8 (11)	16 (34)
5	46 (48)	47 (65)	NA	47 (49)	18 (24)	NA
6	4 (4)	27 (37)	NA	87 (92)	38 (52)	NA
7	68 (72)	48 (66)	22 (46)	24 (25)	17 (23)	23 (48)
8	25 (26)	47 (65)	21 (43)	62 (66)	21 (28)	24 (50)
9	44 (46)	53 (72)	NA	49 (52)	19 (26)	NA
10	40 (42)	41 (56)	NA	54 (56)	29 (40)	NA
11	24 (25)	37 (50)	NA	60 (63)	31 (42)	NA
12	25 (26)	28 (39)	NA	62 (66)	40 (59)	NA
13	60 (63)	57 (78)	NA	31 (33)	15 (20)	NA
14	68 (72)	55 (75)	NA	16 (17)	14 (19)	NA
15	34 (46)	54 (74)	NA	46 (48)	16 (22)	NA

The table shows the distribution of responses to primary questions in the survey among healthcare professionals from hospitals, municipalities and GPs

The response categories 'Strongly agree' and 'Agree' were combined and 'Disagree' and 'Strongly disagree' were combined

The response category 'Not relevant' is not reported in the table

n: Number of responders

GP: General practitioner

NA: Not applicable

Overview of primary questions in the questionnaires

1. It is meaningful that the hospital holds treatment responsibility for 72 h after discharge for patients discharged to municipal nursing care
2. It is my experience that there is widespread awareness of the agreement on 72-h treatment responsibility across sectors
3. I experience that I have been sufficiently prepared by my workplace to use the agreement on 72-h treatment responsibility
4. It is clear to me that, as a general rule, the discharging hospital department should be contacted within 72 h after discharge if a need arises
5. The workload associated with the 72-h treatment responsibility agreement is manageable
6. I feel that the 72-h treatment responsibility agreement allows me to save time in my work
7. The 72-h treatment responsibility agreement has led to changes in workflows at my workplace
8. The 72-h treatment responsibility agreement has led to changes in the way I communicate with colleagues across sectors
9. The 72-h treatment responsibility agreement contributes to better dialogue about the patient across sectors
10. The 72-h treatment responsibility agreement has improved the handover of patients across sectors
11. I experience contacting colleagues from other sectors more frequently than before the introduction of the agreement
12. The 72-h treatment responsibility agreement has increased my understanding of the contexts and options of colleagues in other sectors
13. The 72-h treatment responsibility agreement enables faster support for issues concerning the patient's condition
14. It is my experience that most calls result in a resolution of the given issue
15. The 72-h treatment responsibility agreement has improved continuity in patient care compared with before its implementation

The table shows primary questions in the questionnaires
72-h: 72-hours
