# Supplementary material for: Outcomes of 72-Hour Hospital Treatment Responsibility Post Discharge to Municipal Nursing Care
Source: Int J Integr Care. 2026 Sep 7;26(3):15. doi: 10.5334/ijic.10188 (PMC13564373; doi:10.5334/ijic.10188)
Supplement: Supplementary file 2 [file ijic-26-3-10188-s2.pdf]

REVIEW THE ASSESSMENT FORM IN ADVANCE AND COMPLETE IT BASED ON INFORMATION FROM YOUR OWN SYSTEMS. THE ASSESSMENT FORM SERVES AS A GUIDE FOR PREPARATION FOR THE AUDIT AND AS A GUIDE FOR THE DISCUSSION DURING THE AUDIT MEETING.

First name and age
Patient ID number

Supplementary 2

Questions used for guidance at the audit meetings

When was the patient admitted?

What were the most significant diagnoses?

What treatment has the patient received during the hospitalization?

How many registered calls have there been between the municipality and the hospital?

What was the content of the calls?

How was the issue in the call resolved?

Was a correspondence message sent in connection with the calls?

Was acute or subacute medical follow-up needed within 72 hours?

Is the 72-hour treatment responsibility documented in the discharge report and the discharge summary?

Is the treatment plan in the discharge report clearly stated?

Is the treatment plan in the rehabilitation plan clearly stated?

Is the Shared Medication Record consistent with the prescribed medication dosage?

What treatment and care have the citizen received from the municipality after discharge?

Is there consistency between the services stated in the discharge report and the municipal services the citizen has received?

Have there been any readmissions?

If "yes": Could the readmission have been prevented?

ASSESSMENT OF THE CROSS-SECTORAL EFFORT (NOT TO BE COMPLETED IN ADVANCE)

Is it assessed that the contact has improved the quality of treatment?

Is it assessed that the contact has prevented an unnecessary readmission?

Is it considered that the intervention has created greater continuity of care across sectors? If yes, describe how.

Is it considered likely that the general practitioner could have provided the same intervention? If no, describe why not.

Were there any aspects of the patient pathway across sectors that worked particularly well?

Were there any aspects of the patient pathway across sectors that did not work as well?

Could the municipality or the hospital have done anything during the pathway that would have made the contact unnecessary?

How can the arrangement be adjusted to improve the patient pathway across sectors?
