# Supplementary material for: Outcomes of 72-Hour Hospital Treatment Responsibility Post Discharge to Municipal Nursing Care
Source: Int J Integr Care. 2026 Sep 7;26(3):15. doi: 10.5334/ijic.10188 (PMC13564373; doi:10.5334/ijic.10188)
Supplement: Supplementary file 3 [file ijic-26-3-10188-s3.pdf]

Supplementary 3

Progression in indicators from electronic patient records derived from the Health Platform

Indicators from electronic patient records	Time period where the agreement was fully implemented															
	sep-22	oct-22	nov-22	dec-22	jan-23	feb-23	mar-23	apr-23	maj-23	jun-23	jul-23	aug-23	sep-23	oct-23	nov-23	dec-23
% of 72-hour cases where one or more discharge report is sent	86%	87%	86%	86%	86%	84%	85%	87%	87%	86%	87%	85%	84%	83%	82%	82%
% of correspondence messages sent from hospital to municipality	12%	13%	14%	14%	13%	14%	15%	14%	13%	17%	17%	16%	16%	16%	17%	16%
% of correspondence messages sent from municipality to hospital	4%	4%	5%	5%	4%	5%	5%	4%	4%	4%	5%	5%	5%	4%	4%	5%
% of updated shared medication records at discharge among citizens under the agreement	99%	99%	99%	99%	99%	99%	99%	99%	100%	99%	99%	99%	99%	99%	98%	98%
% of readmissions within 30 days after discharge among citizens under the agreement	24.6%	26.0%	23.20%	24.0%	24.1%	23.8%	23.2%	25.8%	25.2%	25.7%	24.5%	26.5%	24.4%	24.7%	24.9%	24.4%

The table shows how selected indicators from electronic patient records derived from the Health Platform progress from September 2022 where the agreement was fully implemented to December 2023.
